# Asylums Board Report on Antitoxic Serum

**Published:** 1897-05-29

**Authors:** 


### ASYLUMS BOARD REPORT ON ANTITOXIC SERUM.

The medical superintendents of the fever hospitals of the Metropolitan Asylums Board have issued a report on the use of antitoxic serum in the treatment of diphtheria during the year 1896. The total number of cases treated with antitoxin was 2,764, of whom 717 died, giving a case mortality of 25·9 per cent. An examination of these cases, tabulated as they are in the report, brings out two points very strongly—the influence upon the death-rate produced by age and by early treatment. The percentage mortality is seen to progressively diminish from 32·2 in cases under five years of age to 4·2 at ages over twenty, and to rise from 5·2 in those patients who came under treatment on the first day of illness to 31·7 in those who were admitted on the fifth day and after. Even this statement, however, fails to show to the full the gravity of the disease in early life, for, on working out the figures, it is found that among the 253 cases under two years of age which were treated with antitoxin the mortality was at the rate of just over 43 per cent. The question at once arises—How does this death-rate stand in comparison with that among cases not so treated? This question can only be answered by comparing the whole mass of the cases in 1896 (both antitoxin and non-antitoxin cases) with the whole of the cases in 1894, the last year before the introduction of antitoxin. Comparing these two sets of figures, we find that the mortality was 20·8 per cent. in 1896, against 29·6 in 1894, a very considerable gain, when we consider that antitoxin was only administered to 66·2 per cent. of the patients admitted. These, however, were the more severe cases. Perhaps the effect of the treatment can be best judged of by considering only the cases under five years of age. Of these cases 30·2 per cent. died in 1896, against 47·4 per cent. in 1894. It is clear, then, that the benefit of the antitoxin treatment has been especially great among the younger children.

The change in the mortality of the laryngeal complications has also been very marked. The percentage mortality of all laryngeal cases has fallen from 62 in 1894 to 29 6 in 1896; the proportion of these cases which have required tracheotomy has fallen from 56 per cent. to 40·6 per cent.; while the mortality of the operation has fallen from 70·4 per cent. to 42·5 per cent. These are admirable results. In two of the hospitals about 70 per cent. of all tracheotomies have recovered. The proportion of the cases treated with antitoxin at the different hospitals has varied considerably, ranging from 78·8 per cent. at the South-Eastern down to 65·7 per cent. at the Fountain, and then, with a great drop, down to 44·3 per cent. at the North-Western; and it is of some importance to note, as is shown in one of the tables, that this last hospital, in which the antitoxin was administered to so far smaller a proportion of the patients than in the others, was the only hospital in which the diphtheria mortality was higher in 1896 than in 1894. The report deals separately with post-scarletinal diphtheria, and shows that in this formerly fatal condition the mortality has fallen to 5 per cent.

In regard to complications probably connected with the use of antitoxin, it seems that the occurrence of a rash is the most common sequel. It usually takes the form of an urticaria or a vivid patchy erythema, more or less covering the trunk and extremities. It is very similar to the eruptions of measles and septicæmia, is sometimes scarletiniform, and is often accompanied with pyrexia. It appears, however, that with the use of the smaller doses of more potent serum which are now employed the rash, the pains in the joints, and the secondary pyrexia have become comparatively trivial.

The tendency of the whole report is to the effect that in antitoxic serum we have a remedy of greater value than any other in the treatment of diphtheria. It should be added that one of the superintendents declines to subscribe to all the conclusions of the report, although admitting that a reduction in the mortality of the laryngeal cases has actually occurred, and that the percentage of such cases in which tracheotomy had become necessary is reduced.

